# A three-branch 3D convolutional neural network for EEG-based different hand movement stages classification

**DOI:** 10.1038/s41598-021-89414-x

**Published:** 2021-05-24

**Authors:** Tianjun Liu, Deling Yang

**Affiliations:** Key Laboratory of Sustainable Forest Management and Environmental Microorganism Engineering of Heilongjiang Province, Northeast Forestry University, Harbin, 150040 China

**Keywords:** Neuroscience, Neurology

## Abstract

Motor Imagery is a classical method of Brain Computer Interaction, in which electroencephalogram (EEG) signal features evoked by the imaginary body movements are recognized, and relevant information is extracted. Recently, various deep learning methods are being focused on finding an easy-to-use EEG representation method that can preserve both temporal information as well as spatial information. To further utilize the spatial and temporal features of EEG signals, we proposed a 3D representation of EEG and an end-to-end EEG three-branch 3D convolutional neural network, to address the class imbalance problem (dataset show unequal distribution among their classes), we proposed a class balance cropped strategy. Experimental results indicated that there are also a problem of the different classification difficulty for different classes in motor stages classification tasks, we introduce focal loss to address problem of ‘easy-hard’ examples, when trained with the focal loss, the three-branch 3D-CNN network achieve good performance (relatively more balanced classification accuracy of binary classifications) on the WAY-EEG-GAL data set. Experimental results show that the proposed method is a good method, which can improve classification effect of different motor stages classification.

## Introduction

Modern neurophysiological studies shows that the power spectrum of some characteristic frequency components in EEG signals can be changed by actual body movement or imaginary brain movement. The decrease of power spectral ratio is called event-related desynchronization (ERD), and the increase of power spectral ratio is called event related synchronization(ERS)^1, 2^. Brain computer interface (BCI) based on ERS/ERD phenomenon provides a way for communication between computers and human brain by analyzing the electrical signals generated by brain nervous system^3^. Electroencephalogram (EEG) signal is a one of electrical signal widely used in brain computer interface (BCI) systems. A large number of motor imagery classification methods have been proposed. In previous studies, machine learning methods like dynamic connectivity analysis^5^, frequency band analysis^4^, continuous wavelet transform^6^, and Filter Bank Common Spatial Pattern (FBCSP) ^7^ have been widely proposed for EEG decoding. These methods artificially extracted time–frequency features from EEG signals, and then combine these artificially extracted features into feature vectors, which are then used to train classifiers such as support vector machine (SVM)^8, 9^ or decision tree^10^ to classify EEG signals. Therefore, in the above-mentioned methods, the selection of the best filter band is usually subject specific, and it depends heavily on the quality of the hand-made features^11^ thus if the suboptimal frequency band is selected in the feature extraction process, the classification performance may not be the best. Moreover, these methods can not be widely used in large population due to the non universality of subjects.

Compared with machine learning frameworks^12–14^, deep learning methods does not need to extract features manually, and embeds all calculations, including extracting feature and classification, into a single end-to-end network, which can overcome the disadvantages of traditional machine learning^15, 16^. In order to apply deep learning method to MI classification, EEG signals need to be represented as a processable form, which is a prerequisite to be satisfied, to meet this premise, EEG are often represented as a two-dimensional array, which taking the number of sampling electrodes as the height and the time step as the width. A typical method^17^ is to represent EEG signals as 2D images by a short-time Fourier transform (STFT) method. In particular, the spectral content of Mu and beta bands becomes obvious by maintaining the activation mode at different positions, times and frequencies. However, this two-dimensional representation can not keep the spatial information of EEG and the correlation between adjacent electrodes can not be reflected in the two-dimensional array, which leads to the unsatisfactory classification performance of EEG coding. In view of the shortcomings of the above two-dimensional representation methods, to obtain better performance, some more dimensions representation methods were introduced. Zhao et al.^18^ first introduced a three-dimensional representation method of EEG signals, which retains both temporal information and spatial information. Based on this representation, a three-branch 3D CNN is proposed to extract the EEG signal features and complete classification tasks, their architecture achieves an excellent classification performance on BCI competition IV-2a. Compared with the most advanced methods, the performance of this method is significantly improved, indicating that spatial information are important for EEG-based classification tasks.

However, all these methods rarely notice the class imbalance and the different classification difficulty for different classes. In the classification problem, the class imbalance problem (data sets show unequal distribution among their classes) is very common. When the class imbalance is serious, the performance of the model will further get degraded^19^.

To solve the problem of class imbalance, various methods have been designed to obtain a more practical classification model, the most common method is to use resampling techniques (for example, oversampling and undersampling) to modify the class distribution of the training set and make it more balanced, thereby allowing conventional learning algorithms to perform well^20–24^. Another popular method is cost-sensitive learning, which allocates higher cost when misclassifying a minority class instances at the algorithm level^25, 26^, or using SMOTE (synthetic minority oversampling technology) and its variants^27–30^ to generate synthetic minority samples. However, SMOTE have difficulties in processing high-dimensional data^31^. Another method is to weight the training samples based on the class imbalance in the optimization function of the classifier^32^. To further address this problem, Su et al.^33^ proposed four methods to overcome the problem of class imbalance, They tested these methods and three types of unbalanced EEG classification problems, and observed significant improvements.

Class imbalance is addressed by a two-stage cascade and sampling heuristics in object detection. The proposal stage (e.g., RPN^37^, Selective Search^34^, DeepMask^36^, EdgeBoxes^35^) reduce the objects to a smaller number (for example, 1–2 k), and filter out a large number of background samples. In the second stage, to keep a acceptable balance between background and foreground, sampling heuristics are implemented^38^. The two-stage detection method can achieve very high results, but it also has a big disadvantage: time-consuming. To reduce the time-consuming while not reducing the detection effect, a one stage object detector^39^ have been presented to matches the state-of-the-art COCO AP of more complex two-stage detectors. They suggest that the standard cross entropy loss should be reshaped to solve this kind of imbalance, so as to reduce the weight of the loss assigned to well classified examples. Focal loss can also be migrated to other classification tasks with class imbalance.

In this study, similar to method in^18^, a 3D representation of EEG signal is introduced, which preserves both temporal and spatial information, on this basis, we designed a three-branch 3D CNN to complete feature extraction and classification. One of the primary contributions of the proposed framework is that a class equal cropped strategy are proposed for the WAY-EEG-GAL data set(a class imbalance datasets). At the same time, we think that there are not only a problem of class imbalance in EEG classification, but also a issue of ‘easy-hard’ example(the different classification difficulty for different classes). So Another contribution of the proposed method is that we introduce the focal loss to address this problem and achieve good performance (more balanced results of binary classifications) on the WAY-EEG-GAL data set. In addition, The proposed methods were evaluated on the BCI competition IV 2a datasets(a well-balanced dataset) to verify the effectiveness of our proposed framework on well-balanced dataset.

## Methods

In the following sections, we will describe 3D representation of EEG, three-branch 3D CNN, focal loss and classification strategy.

### 3D representation of EEG

Zhao et al.^18^ designed a three-dimensional model of EEG. Firstly, according to the distribution of the sampling electrodes, the EEG signal is converted into a two-dimensional array, and then the points without electrodes are filled into 0. After that, this 2D array was expanded to 3D array by using temporal information of EEG signals. In this study, we designed a 3D representation of EEG similar to method in Ref.^18^.

**Figure 1:**
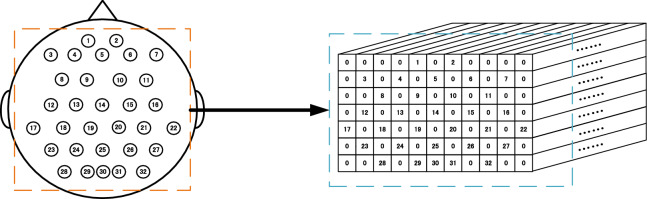
EEG signal 3D representation for the WAY-EEG-GAL datasets. Left: Sampling electrodes spatial distribution. Right: 3D representation. (7 × 11 × 480 array) of EEG.

At the same time, because of the insensitivity of the network proposed in this study to the filtering, we does not use the filtering method, but only does the subtraction average value processing to improve the classification effect of the network. In this study, since the EEG signal of the WAY-EEG-GAL dataset equipment has 32 sampling electrodes with spatial distribution according to the international 10–20 system, the same representation method is also to represent the EEG signal of these 32 channels. The specific representation process is shown in Fig. 1.

### The adjusted three-branch 3D CNN

**Figure 2:**
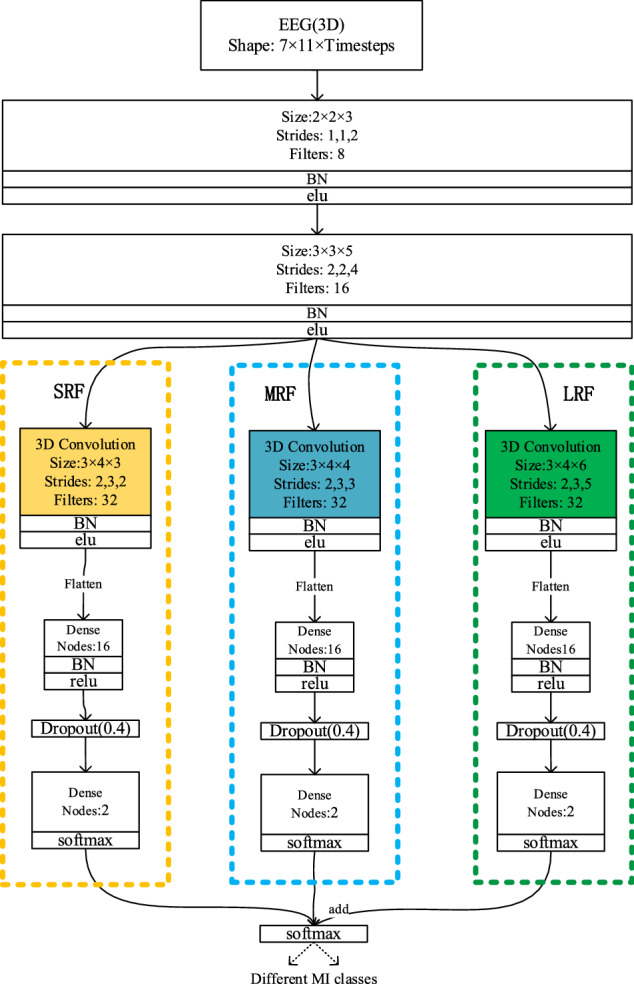
The architecture of three-branch 3D-CNNs.Three branch networks are, respectively, framed by three dashed boxes with different colors, and the input of different convolution layers is distinguished by connecting lines of different colors, ‘Dropout(0.4)’ means dropout method( dropout percentages = 0.4).

Based on the 3D representation of EEG, a three-branch 3D CNN is also used to classify the motion intention in different stages. However, because in this study, our research is based on binary MI Classification method, the three-branch 3D CNN used in this section has been adjusted on the basis of Ref.^18^. The adjusted network is shown in Fig. 2.

As can be seen from Fig. 2 in Ref.^18^ and Fig. 2 in this paper. The adjustment of the model includes the following points. Firstly, the overall structure and parameters of the model are adjusted. Secondly, in this study, the problem of classification is changed from four-classes MI Classification to binary MI Classification, that is, the number of fully connected network nodes in the penultimate layer of the network is reduced from 4 to 2, at the same time, due to the reduction of classification classes, the number of nodes in other full connection layers is reduced. Thirdly, to prevent over-fitting problem, we introduce dropout method(dropout values = 0.4) described in^40^ at the full connection layer(The optimal dropout percentages are obtained by many experiments with different dropout percentages range from 0.3 to 0.7 with an interval of 0.1).

### Focal loss

The focal loss is designed to address the one-stage object detection tasks^41^. In this study, we introduce focal loss to address problem of ‘easy-hard’ example.

To understand the focal loss function clearly, starting from the cross entropy (CE) of binary classification, we introduce the focal loss.

1 $${\text{CE}}\left( {p,y} \right) = \left\{ {\begin{array}{*{20}l} {\begin{array}{*{20}l} { - \log \left( p \right)} \hfill & {{\text{ if }}y = 1} \hfill \\ \end{array} } \hfill \\ {\begin{array}{*{20}c} { - \log \left( {1 - p} \right)} & {{\text{otherwise}}} \\ \end{array} } \hfill \\ \end{array} } \right.$$

$y$ ∈ {± 1} allocates the ground-truth class in the above and $p$ ∈ [0, 1] is the model’s estimated probability for the class with label $y$ = 1. For convenience, we define $p_{t}$:

2 $$p_{t} = \left\{ {\begin{array}{*{20}l} {\begin{array}{*{20}l} p \hfill & {{\text{ if }}y = 1} \hfill \\ \end{array} } \hfill \\ {\begin{array}{*{20}c} {1 - p} & {{\text{otherwise}}} \\ \end{array} } \hfill \\ \end{array} } \right.$$

and rewrite it as ${\text{CE}}\left( {p,y} \right) = {\text{CE}}\left( {p_{t} } \right) = - \log \left( {p_{t} } \right)$.

A notable feature of this loss is that even the easy examples ($p_{t}$ ≫ . 5) will result in non-trivial loss. These small loss values can overwhelm the less class when summed over a large number of easy examples. In this study, focal loss is introduced to address the problem of ‘easy-hard’ example. Focal loss is a function that add a modulating factor $\mathop {(1 - \mathop p\nolimits_{t} )}\nolimits^{\gamma }$ to the cross entropy loss, $\gamma \ge {0}$, is a tunable *focusing* parameter.

The focal loss had been defined as:

3 $${\text{FL}}\left( {p_{t} } \right) = - \left( {1 - p_{t} } \right)^{\gamma } \log \left( {p_{t} } \right)$$

Focal loss have two properties. 1) When an example is misclassified and $p_{t}$ is small, the modulating factor is near 1 and the loss is unaffected. As $p_{t}$ → 1, the factor goes to 0 and the loss for well-classified examples is down-weighted, so model will pay more attention to hard example. 2) The focusing parameter γ smoothly adjusts the rate at which easy examples are down-weighted. When γ = 0, FL is equivalent to CE, and as γ is increased the effect of the modulating factor is likewise increased(We found that each binary classification task has its γ value to work best in our experiments).

Furthermore, we can also slightly improve the model recognition effect by adding α-balanced variant, note that adding only $\alpha_{t}$ can balance the importance of negative and positive samples, but it can not address the problem of “easy-hard” examples. To ensure the loss value not too small to stop the training, we multiply the formula by one thousand, just like this:

$${\text{FL}}\left( {p_{t} } \right) = - \alpha_{t} \left( {1 - p_{t} } \right)^{\gamma } \log \left( {p_{t} } \right) \times 1000$$

Intuitively, the modulating factor reduces the loss contribution from easy examples and extends the range in which an example receives low loss. For example, if the γ = 2, an example classified with *p* = 0.9 would have 100× lower loss compared with CE and with ≈ 0.968 it would have 1000× lower loss. However, the loss of the sample with a prediction probability of 0.3 is relatively large. When the prediction probability is 0.5, the loss is only reduced by 0.25 times, so model pay more attention to the hard example. In this way, the influence of easy example is reduced.

In this study, we use α to balance sample size, α have been defined in this study:

5 $$\mathop \alpha \nolimits_{{}} = \frac{{num\_c{2}}}{(num\_c1 + num\_c2)}$$

6 $$\alpha { = }\left\{ {\begin{array}{*{20}c} \alpha & {y = 1} \\ {1 - \alpha } & {y = 0} \\ \end{array} } \right.$$

In Eq. (5), *num_c*1 is the amount of class 1, and *num_c2* is the amount of class 2.

### Classification strategy

#### Cropped strategy

Cropped training has been applied to the image recognition field for increasing the training data and improving the training effect^42, 43^. In Ref.^18^, they adopted a cropped training approach for EEG 3D representation by sliding a 3D window which covers all sampling electrodes on each EEG data trial along the time dimension with a data stride 1, in this way, they obtained more training data, in this study, we use the same cropped method as in^18^. We first extract the EEG data with a length of 500, and then cropped it with a length of 480, through this approach, the cropped strategy will generate some cropped data just as Table 1, Note that the amount of training data we got is unbalanced.

**Table 1:** The amount of training data of four motion stages obtained by cropped strategy.

Subject ID	C1	C2	C3	C4
S1	5512	196,238	5560	5295
S2	52,032	276,729	6327	14,673
S3	6065	186,403	5487	5530
S4	6278	201,843	8543	8792
S5	27,890	228,718	6202	7210
S6	26,462	233,637	6147	14,712
S7	78,849	239,767	21,997	26,298
S8	10,791	217,182	5676	6036
S9	27,349	210,387	7009	14,817
S10	25,707	251,869	8154	9750
S11	56,070	233,336	7971	19,627
S12	51,158	247,668	19,423	41,810

“Sx” means “Subject x”, “Cx”means different class “class x”.

Table 1 shows that the cropped data of each experimenter is different and unbalanced, and the mount of cropped data in the second stage is generally more than that of other stages.

In order to balance the training data set, another data cropped method is also proposed. The adjustment method follows the principle of keeping the data amount of each class around 6000 or less, there are two different cropped methods for different situations.

If the amount of cropped data for some classes is still less than 6000 when clipping at a cropped step with 1, we would crop this data with 1, based on amount of this data, crop another class. That is, the cropped step size can be calculated like this:

7 $${\text{step}} = \frac{num\_c1}{{num\_c2}}$$

*“num_c1”* is the amount of class more than 6000, *“num_c2”* is the amount of another class less than 6000.

If the amount of data for both classes is more than 6000 when clipping at a cropped step with 1, we wound keep the amount of cropped data around 6000 through clipping them with step like Eqs. (8) and (9).

8 $${\text{step1}} = \frac{num\_c1}{{3000}}$$

9 $${\text{step2}} = \frac{num\_c2}{{3000}}$$

*“num_c1”* is the amount of the class 1, *“num_c2”* is the amount of the another class.

Note that this cropped method just can ensure that the training data of each class is approximately equal rather than completely equal. The purpose of this method is to balance different kinds of EEG data to achieve a better classification effect .

#### Network optimization

When it comes to network optimization, similar to the earlier work^18^, all weights, as well as the initial value, are initialized using the normalized initialization method in Ref.^39^, and the learning rate is 0.01. The negative log-likelihood cost has been adopted as the optimization criterion^44^, and the optimization method uses ADAM with default parameter values described in Ref.^45^. In the training process, if the cost does not reduce within 20 epochs, the training will be stopped, and the network weight with the lowest cost will be restored from the epoch.

## Experiment and results

### EEG data

The WAY-EEG-GAL is not only the first but also the only published data set of brain wave signals related to different stages of action identification. The EEG data in this data set includes all the EEG data in the whole process of experimental paradigm.For EEG signal recording, 32 EEG sampling electrodes are used, which meet the international 10–20 standard. The EEG sampling electrode continuously samples the EEG signal in the process of each sub-experiment with a sampling frequency of 500 Hz. In terms of time point recording of experimental data, the data set provides 43 time point information such as the start time of each sub-experiment, the time when the LED indicator lights up, and the time when the LED indicator lights out. Through these time point information, we can map the brain wave signal data with different events one by one. These time point information are all placed in the human joints or moved from sensors on the surface of the animal. A complete description of the WAY-EEG-GAL data set is available in Ref.^46^.

Because the purpose of this study is to identify the movement intention in different stages of the action, this study extracts four EEG data in 3936 * 12 sub-experiments of all 12 subjects by using the time information and brain wave signal data of these several time points. The definition of EEG data of four different motion stages is shown in the Table 2.

**Table 2:** The definition of EEG Data of four different motion stages.

Action state	Meaning of time period	Start time	End time
First movement state	Hands start to move	tHandStart	tBothStartLoadPhase
Second movement state	Finger start to apply load force	tBothStartLoadPhase	LEDOff
Third movement state	Fingers apply load and hands return objects	LEDOff	tReplace
Fourth movement state	Return the hand to its original position	tReplace	tHandStop

In this study, in consideration of the relationship between the stages of the action, we transfer four-classes MI classification experiments to three continuous binary classification experiments. Note that this cropped method just can ensure that the training data of each class is approximately equal rather than completely equal. The purpose of this method is to balance different kinds of EEG data to improve the classification effect of the model. For the evaluations using cross-validation of the WAY-EEG-GAL datasets, the training and testing datasets are combined and then randomly divided into nine subsets of equal size, which eight subsets were used as training data and a single subset was used as the testing data in each run.

The BCI competition IV 2a dataset consists of EEG data from 9 subjects, using 22 Ag/AgCl electrodes to record the EEG signals. Each subject recorded two sessions on different days and the recorded signals were sampled with 250 Hz. The recorded signals were sampled with 250 Hz and bandpass-filtered between 0.5 Hz and 100 Hz. A single run consisted of 48 trials, which yielded 288 trials per session. The duration of each trial consisted of a fixed period of 2 s and a reminder period of 1.25 s, followed by a period of 4 s of motor imagery. More details on the datasets are available in Ref.^47^. For this dataset, we adopt a same cropped strategy in Ref.^18^.

In the presented study^18^, a 1.25 s period of EEG data is chosen as the experimental data, after the visual cue in each trial. These are further represented as 3D representation without any preprocessing. The sampling frequency is 250 Hz, so 313 sampling points can be generated in 1.25 s sampling time. It can be concluded from the results of Ref.^18^ that for the EEG signal with 250 Hz sampling frequency, the EEG signal with 240 sampling points has covered the features related to motor imagery.

### Classification performance by different model’s depth and branch

#### Comparison of different model depths

In Ref.^17^, three different depths CNNs are used to do EEG signals classification. Experimental results show that the depth of CNN has a remarkable impact on classification effect, and the classification effect of shallow CNN is better than that of deep CNN. To find the best appropriate model depth, we changed the depth of the model to what the Tables 3 and 4 shows, and then compare the classification effects. The network shown in Table 3 is shallower than the proposed network, on the contrary, the model shown in Table 4 is deeper than our proposed model.

**Table 3:** The three-branch 3D CNN with two convolutional layer.

Convolutional Layer num	Branch type	SRF	MRF	LRF
1st	Shape	2 × 2 × 3	2 × 2 × 3	2 × 2 × 3
	Stride	1 × 1 × 2	1 × 1 × 2	1 × 1 × 2
2nd	Shape	3 × 4 × 5	3 × 4 × 7	3 × 4 × 9
	Stride	2 × 3 × 4	2 × 3 × 6	2 × 3 × 8

**Table 4:** The three-branch 3D CNN with four convolutional layer.

Convolutional Layer num	Branch Type	SRF	MRF	LRF
1st	Shape	2 × 2 × 3	2 × 2 × 3	2 × 2 × 3
	Stride	1 × 1 × 2	1 × 1 × 2	1 × 1 × 2
2nd	Shape	3 × 3 × 5	3 × 3 × 5	3 × 3 × 5
	Stride	2 × 2 × 4	2 × 2 × 4	2 × 2 × 4
3rd	Shape	2 × 2 × 1	2 × 2 × 3	2 × 2 × 5
	Stride	1 × 1 × 1	1 × 1 × 2	1 × 1 × 4
4th	Shape	2 × 3 × 1	2 × 3 × 3	2 × 3 × 5
	Stride	2 × 3 × 1	2 × 3 × 2	2 × 3 × 4

We completed the experiment with cropped strategy but without Focalloss on the WAY-EEG-GAL dataset, by comparing classification effect shown in Table 5 of three different network mentioned above with each other, it can be found that except for the c3&c4 experiment, our proposed three-branch 3D CNN perform best in all binary classification experiments. This indicates that if the model depth is too shallow, it will not extract features very well, and if the model is too deep, it will result in over-fitting to reduce slightly the training effect, so our proposed network’ depth is the most appropriate depth to achieve the best classification effects.

**Table 5:** Results of ninefold cross-validation training corresponding to different depth of network.

Experiment	Class	3D CNN type	ACC_mean
C1&C2	C1	Shallow 3D CNN	0.770
		Our proposed 3D CNN	0.771
		Deeper 3D CNN	0.767
	C2	Shallow 3D CNN	0.606
		Our proposed 3D CNN	0.635
		Deeper 3D CNN	0.630
C2&C3	C2	Shallow 3D CNN	0.903
		Our proposed 3D CNN	0.939
		Deeper 3D CNN	0.917
	C3	Shallow 3D CNN	0.525
		Our proposed 3D CNN	0.595
		Deeper 3D CNN	0.530
C3&C4	C3	Shallow 3D CNN	0.685
		Our proposed 3D CNN	0.686
		Deeper 3D CNN	0.662
	C4	Shallow 3D CNN	0.771
		Our proposed 3D CNN	0.785
		Deeper 3D CNN	0.789

#### Comparison of the different number of network’s branches

**Figure 3:**
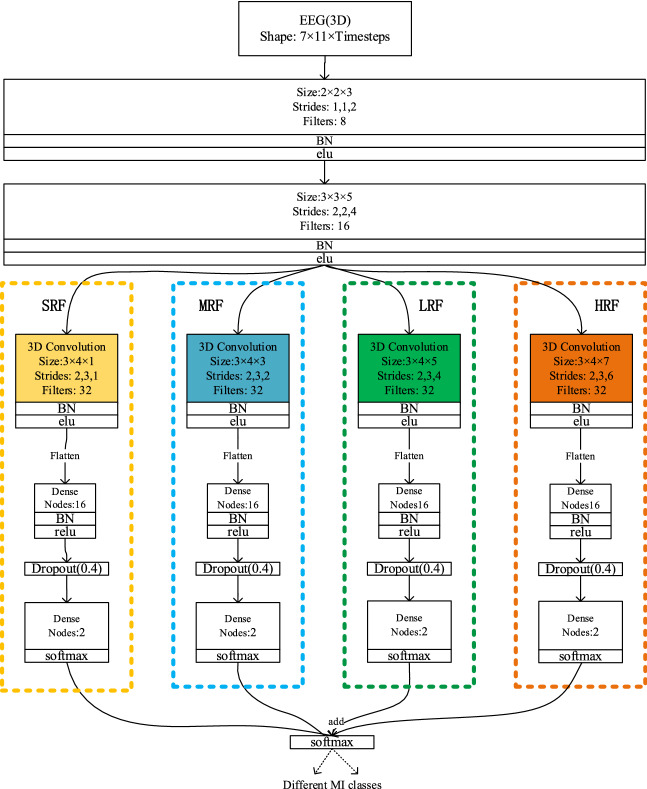
The architecture of four-branch 3D-CNNs. Three branch networks are, respectively, framed by three dashed boxes with different colors, and the input of different convolution layers is distinguished by connecting lines of different colors.Dropout(0.4)’means dropout method( dropout percentages = 0.4).

In this section, To further explore the influence of the number of branches on the classification accuracy, a set of experiments has been carried out on three networks with a different number of branches, which are, respectively composed of SRF and MRF, and our proposed three-branch 3D CNN, and a more complex four-branch network just like Fig. 3. We can observe that in total, the proposed 3D CNN can reach higher accuracy than the two-branch network, and achieved a probably similar accuracy to complex network shown on Table 6, but the complex network also has a big disadvantage: more parameters and more time consuming, this means, the three-branch network is more effective than other multi-branch network for the WAY-EEG-GAL dataset.

**Table 6:** Results of ninefold cross-validation training corresponding to different number of network branch.

Experiment	Class	3D CNN type	ACC_mean
C1&C2	C1	Two-branch	0.747
		Three-branch	0.771
		Four-branch	0.769
	C2	Two-branch	0.601
		Three-branch	0.635
		Four-branch	0.621
C2&C3	C2	Two-branch	0.904
		Three-branch	0.939
		Four-branch	0.934
	C3	Two-branch	0.527
		Three-branch	0.595
		Four-branch	0.596
C3&C4	C3	Two-branch	0.647
		Three-branch	0.686
		Four-branch	0.681
	C4	Two-branch	0.773
		Three-branch	0.785
		Four-branch	0.789

ACC_mean means the mean accuracy of all subjects.

### Influence of focal loss

In the previous section, experimental shows that after using the data equal cropped strategy, we get the more balanced training data, but there are still a big gap in test accuracy between two different classes. Therefore, we introduce the focal loss function when two classes test accuracy gap is greater than 0.3 and use the same training strategy as proposed above. We try with γ = 0–11(step size is 0.5) to obtain the best accuracy and corresponding γ value.

**Figure 4:**
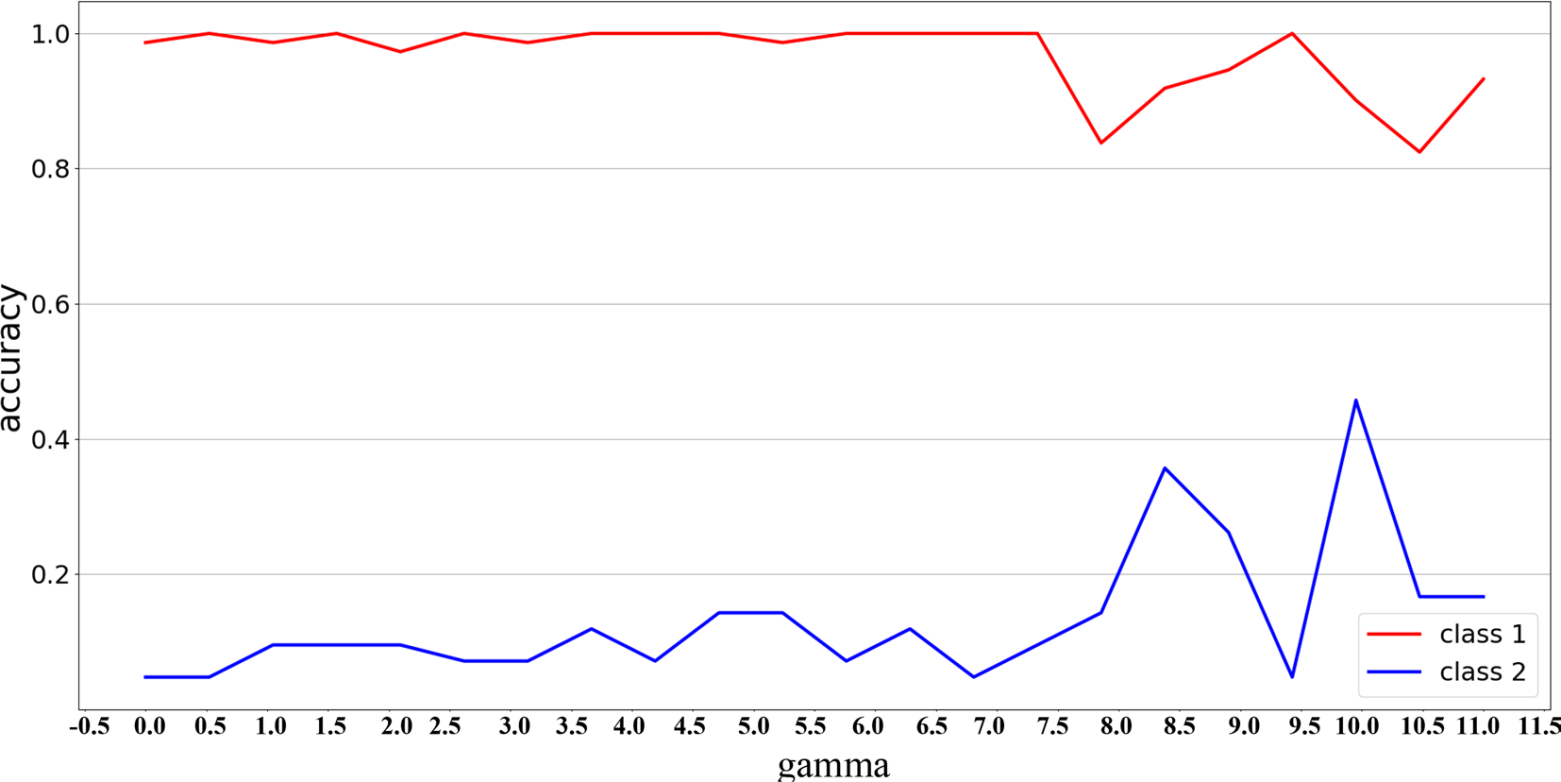
The binary classification (the first and second stages in object2.) test accuracy corresponding to different γ values.

As shown in Fig. 4, when the class test accuracy of framework trained with CE function is extremely imbalanced(class1: 1.000, class2: 0.043), how the test accuracy changes as the γ value increases. When γ is between 0 and 7.5, with the γ increase, the accuracy of class 2 fluctuates below 0.2, while the accuracy of class 1 does not change much. When γ is between 7.5 and 10.0, the accuracy of class1 decrease to about 0.9, while the accuracy of class2 increases with a larger value(about 0.3). At the same time, the accuracy has been fluctuating in the middle of a relatively considerable value. From the Fig. 4, we found the optimal value(class1: 0.907, class2: 0.435) when γ = 10.0, then we obtained the final accuracy by averaging thirty results with γ = 10.0, in this way, we obtained all the binary classification accuracy in Table 7.

**Table 7:** Results of ninefold cross-validation training with focal loss function.

Subject ID	Binary experiment no.	ACC			
		With CE		With focal loss	
S2	C1&C2	1.0	0.043	0.897	0.495
	C2&C3	1.0	0.245	0.913	0.422
	C3&C4	0.206	0.959	0.343	0.885
S3	C2&C3	0.865	0.382	0.833	0.529
S5	C1&C2	0.949	0.452	0.920	0.591
	C2&C3	0.929	0.463	0.912	0.622
S9	C1&C2	0.259	0.981	0.520	0.888
	C2&C3	0.981	0.283	0.892	0.430
	C3&C4	0.550	0.910	0.646	0.873
S10	C1&C2	0.802	0.500	0.753	0.652
	C2&C3	1.000	0.132	0.978	0.378
S11	C1&C2	0.908	0.294	0.801	0.497

**Figure 5:**
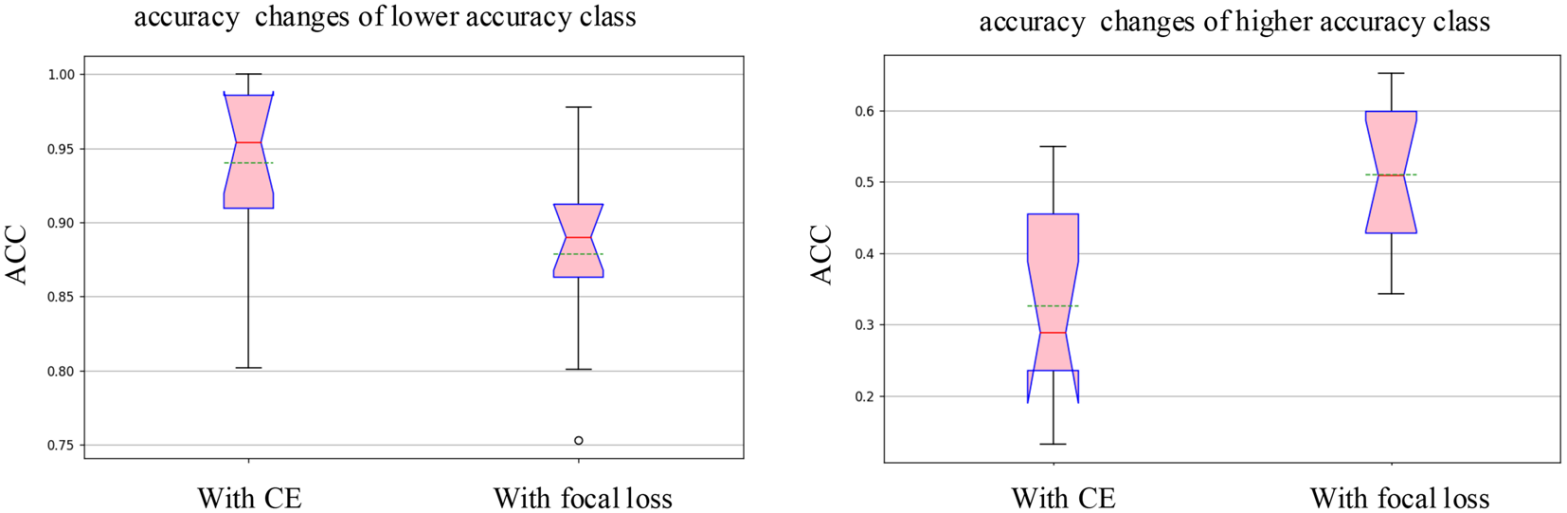
Change of test accuracy of higher and lower accuracy classes before and after introducing focal loss.

The box-plot of Fig. 5 shows the accuracy distribution of all experiments before and after the introduction of focal loss. From Table 7 and Fig. 5, it can be seen that after the introduction of focal loss, the classification effect is improved, and the classification accuracy is mostly above 0.4. However, with the accuracy of class with low test accuracy(hard example) in experiment with CE increase, that of class with high test accuracy(easy example) in experiment with CE decrease slightly, just as Fig. 5, we think this is because focal loss makes the model pay more attention to hard example, but the average decrease value(about 0.06) is far less than the average increase value (0.22), so we think focal loss function improves the classification effect of the framework.

We obtained the final test accuracy after training with focal loss on test accuracy imbalance experiment shown in Table 8. These results indicated that focal loss can indeed improve the EEG decoding performance.

**Table 8:** Comparison of mean classification accuracy of all subject before and after introducing Focaloss.

Experiment	Class	ACC before introducing FL	ACC after introducing FL
C1&C2	C1	0.771	0.747
	C2	0.635	0.698
C2&C3	C2	0.939	0.918
	C3	0.595	0.669
C3&C4	C3	0.686	0.705
	C4	0.785	0.776

### Overall comparison

In this section, the proposed methods were evaluated on the WAY-EEG-GAL(a class unbalanced dataset) and the BCI competition IV 2a datasets(a well-balanced dataset) to verify the effectiveness of our proposed framework on class unbalanced dataset as well as well-balanced dataset.

Here Cohen’s kappa coefficient^48^ is used to evaluate the performance of different networks on the BCI IV 2a(It is also used to measure the classification effect in later section). The kappa values reported in Table 9 are all averaged over 50 results using different model initialization. Kappa value is defined as (10) where $P_{0}$ is the proportion of observed agreement and $P_{e}$ is the probability that agreement is due to chance. And we use mean classification accuracy to evaluate the performance of different networks on the WAY-EEG-GAL datasets. The mean values of 12 subject reported in Table 10 are all averaged over 50 results using different model initialization.

10 $$K{\text{a}}ppa = \frac{{P_{0} - P_{e} }}{{1 - P_{e} }}$$

**Table 9:** Comparison of three state of the art MI classification methods with our proposed 3D CNN on the BCI IV 2a.

Subject ID	Our proposed 3D CNN	FBCSP	C2CM	Multi-branch 3D CNN
1	0.675	0.68	0.833	0.699
2	0.463	0.42	0.537	0.459
3	0.794	0.75	0.870	0.788
4	0.604	0.48	0.556	0.594
5	0.644	0.40	0.5	0.647
6	0.551	0.27	0.273	0.538
7	0.647	0.77	0.861	0.653
8	0.693	0.76	0.778	0.702
9	0.731	0.61	0.727	0.713
Mean	0.645	0.571	0.659	0.644
SD	0.098	0.206	0.204	0.100

**Table 10:** Comparison of three state of the art MI classification methods with our proposed 3D CNN on the WAY-EEG-GAL datasets.

Experiment	Class	3D CNN type	ACC_mean
C1&C2	C1	FBCSP	0.981
		C2CM	0.876
		Multi-branch 3D CNN	0.869
		Our proposed 3D CNN	0.747
	C2	FBCSP	0.245
		C2CM	0.304
		Multi-branch 3D CNN	0.267
		Our proposed 3D CNN	0.698
C2&C3	C2	FBCSP	0.956
		C2CM	0.935
		Multi-branch 3D CNN	0.994
		Our proposed 3D CNN	0.918
	C3	FBCSP	0.314
		C2CM	0.298
		Multi-branch 3D CNN	0.216
		Our proposed 3D CNN	0.669
C3&C4	C3	FBCSP	0.413
		C2CM	0.298
		Multi-branch 3D CNN	0.362
		Our proposed 3D CNN	0.705
	C4	FBCSP	0.855
		C2CM	0.869
		Multi-branch 3D CNN	0.815
		Our proposed 3D CNN	0.776

Three state of the art MI classification methods in the literature and compared these methods with our proposed 3D CNN are introduced in Tables 9 and 10.

We briefly introduce three state of the art algorithms.

FBCSP: FBCSP^7^ is a two-stage method. Firstly, they adopt a group of band-pass filters and CSP algorithm to extract the optimal spatial features from a specific frequency band, and then the classifier is trained to classify the extracted features.

C2CM: C2CM^49^ first uses FBCSP as data preprocessing method, and then uses CNN to extract features. The performance of this method is better than that of FBCSP, but there are trouble, it is difficult to change the parameters according to different objects.

Multi-branch 3D CNN: Multi-branch 3D CNN^18^ is a deep learning framework with three branch 3D CNN, where each branch has a distinct receptive field. Based on the previous studies, the Multi-branch 3D CNN is considered to be a state-of-the-art classification method on the BCI IV 2a.

The experiment was carried out on the BCI IV 2a with cross entropy (CE) thanks to its balanced class. It can be seen from Table 9 that our network has achieved the same effect as Multi-branch 3D CNN^18^, because the depth of our network is the same as its depth, which is three convolution layers to extract the features of EEG signal. At the same time, our network is better than FBCSP in classification effect and C2CM in robustness, which effectively shows that our network has good classification effect on well balanced dataset.

In order to further demonstrate better classification performance of our proposed network on class unbalanced dataset, we completed experiment on WAY-EEG-GAL datasets with our proposed cropped strategy and Focalloss and then compared the effectiveness of our network with other state of the art MI classification methods. Table 10 compared the classification results of our proposed network with other state of the art networks, these networks can't solve the problem of class imbalance in binary MI Classification(The accuracy of one class is much higher than the other), just like FBCSP in experiment C1&C2, the accuracy of C1 is much higher than that of C2 due to class imbalance and ‘easy-haed’ example, in contrast, thanks to our cropped strategy and Focalloss function, our proposed network can solve these two problems well to obtain better and more balance classification affect.

## Discussions

### Multi-branch architecture

Many deep learning studies for EEG classification such as Multi-branch 3D CNN^18^ have used multi-branch structure, Zhao et al.^18^ compared the classification effect of three single-branch 3D CNN with multi-branch 3D CNN and verified the advantages of a multi-branch framework. In this study, classification effect of two-branch 3D CNN, three-branch 3D CNN and four-branch 3D CNN were compared. Experimental results shows that with the increase of network branches, the classification effect can be improved to a certain extent, but it will inevitably increase the complexity of the network to increase the training time, so it is necessary to find a suitable number of branches according to the actual situation such as computational power and time limit for BCI equipment.

### Extreme imbalance problem

we adapt a cropped strategy to address class imbalance problem, but there are still a ‘easy-hard’ problem, and we introduce focal loss to solve this problem because of its two properties. (1) When an example is misclassified and $p_{t}$ is small, the modulating factor is near 1 and the loss is unaffected. As $p_{t}$ → 1, the factor goes to 0 and the loss for well-classified examples is down-weighted, so model will pay more attention to hard example. (2) The focusing parameter γ smoothly adjusts the rate at which easy examples are down-weighted. From the experiment and results part, we can see that there will be an appropriate γ for different subject. We can find the best γ value through a large number of experiments to optimize our classification effect. In this work, we don't rely entirely on focal loss to solve the all of problem. For the extreme imbalance problem, we may need a combination of various methods to solve this problem. In this study, we first use the cropped strategy to balance the amount of data, and then address focal loss to solve the "easy hard" problem. In the field of machine learning, class imbalance is always a trouble. In order to solve this problem, maybe we can use more methods such as expanding data or combination of these methods to solve this problem in the future work.

### Limitation and future work

Although our research has solved the class imbalance problem to a certain extent, there are still some room for improvements. (1) 3D representation, our proposed 3D presentation pads the no electrode point with 0, which has no features of EEG signals, maybe we can use other padding methods which contains the features of all the electrode signals instead of this one to make full use of the 3D representation. (2) 3D CNN structure. A large number of studies have proved that deeper network can extract features better. In general, our proposed 3D CNN can achieve a better classification effect, we find that the classification effect of the network with three convolution layers is better than that of the network with two convolution layers, but adding another convolution layer do not improve the classification effect of the network. This shows that the current network structure can not simply improve the classification performance by increasing the depth of the network. Maybe we can get inspiration from these state of the art deep networks such as ResNet^50^ and Densenet^51^, and improve the network structure of 3D CNN to increase the depth of network to achieve a better classification performance.

## Conclusions

In this work, we proposed a three-branch 3D convolutional neural network with a class equal cropped strategy for class imbalance problem to tackle hand movement stages classification tasks. In addition, to address problem of ‘easy-hard’ examples, we introduce focal loss and adjust slightly it to meet our experiment, after that, we got more balanced and high test accuracy on the WAY-EEG-GAL data set, which shows that focal loss can address the problem of ‘easy-hard’ example well in the EEG classification tasks. The proposed method is user-friendly and can be applied to other MI classification tasks as an effective method.

## References

[1] Pfurtscheller G, Da Silva FHL (1999). Event-related EEG/MEG synchronization and desynchronization: basic principles. Clin. Neurophysiol..

[2] Tang Z, Sun S, Zhang S (2016). A brain-machine interface based on ERD/ERS for an upper-limb exoskeleton control. Sensors.

[3] He L, Hu D, Wan M, Wen Y, von Deneen KM, Zhou M (2016). “Common Bayesian network for classification of EEG-based multiclass motor imagery BCI. IEEE Trans. Syst. Man Cybern. Syst..

[4] Wang Y, Veluvolu KC, Lee M (2013). Time-frequency analysis of band-limited EEG with BMFLC and Kalman filter for BCI applications. J. Neuroeng. Rehabil..

[5] Li Y, Lei M-Y, Cui W, Guo Y, Wei H-L (2019). A parametric time frequency-conditional granger causality method using ultra-regularized orthogonal least squares and multiwavelets for dynamic connectivity analysis in EEGs. IEEE Trans. Biomed. Eng.

[6] Li Y, Cui WG, Luo ML, Li K, Wang L (2017). High-resolution time-frequency representation of EEG data using multi-scale wavelets. Int. J. Syst. Sci..

[7] Ang, K. K., Chin, Z. Y., Zhang, H. et al. Filter bank common spatial pattern (FBCSP) in brain-computer interface. In *2008 IEEE International Joint Conference on Neural Networks (IEEE World Congress on Computational Intelligence*, 2390–2397 (2008).

[8] Wu W, Chen Z, Gao X, Li Y, Brown EN, Gao S (2015). Probabilistic common spatial patterns for multichannel EEG analysis. IEEE Trans. Pattern Anal. Mach. Intell..

[9] Wang L (2017). Automatic epileptic seizure detection in EEG signals using multi-domain feature extraction and nonlinear analysis. Entropy.

[10] Ma L, Gu L, Li B, Ma Y, Wang J (2015). An improved K-means algorithm based on mapreduce and grid. Int. J. Grid Distrib. Comput..

[11] Zhihui F (2016). Research on the prediction of the e-commerce profit based on the improved parallel PSO-LSSVM algorithm in cloud computing environment. Int. J. Grid Distrib. Comput. NADIA.

[12] Li Y, Song H, Zhang G, Chen D, Wang Z, Cui P (2016). Improvement of SVM image reconstruction algorithm in ECT system. Int. J. Grid Distrib. Comput..

[13] Cui J, Liu B, Wang G, Mingyue Y, Gao Y (2015). Life trend analysis of aircraft’s key component based on power spectral envelope energy and SVM. Int. J. Adv. Sci. Technol..

[14] Majidnezhad V, Kheidorov I (2013). The SVM-based feature reduction in vocal fold pathology diagnosis. Int. J. Fut. Gener. Commun. Netw..

[15] Min S, Lee B, Yoon S (2016). Deep learning in bioinformatics. Brief Bioinform..

[16] Li Y, Cui W, Luo M, Li K, Wang L (2018). Epileptic seizure detection based on time-frequency images of EEG signals using Gaussian mixture model and gray level co-occurrence matrix features. Int. J. Neural Syst..

[17] Schirrmeister RT, Springenberg JT, Fiederer LDJ (2017). Deep learning with convolutional neural networks for EEG decoding and visualization. Hum. Brain Mapp..

[18] Zhao X, Zhang H, Zhu G, You F, Kuang S, Sun L (2019). A multi-branch 3D convolutional neural network for EEG-based motor imagery classification. IEEE Trans. Neural Syst. Rehabil. Eng..

[19] Yang Q, Wu X (2006). 10 challenging problems in data mining research. Int. J. Inform. Technol. Decis. Mak..

[20] Cateni S, Colla V, Vannucci M (2014). A method for resampling imbalanced datasets in binary classification tasks for real-world problems. Neurocomputing.

[21] Chawla NV, Bowyer KW, Hall LO (2002). SMOTE: synthetic minority over-sampling technique. J. Artif. Intell. Res..

[22] Estabrooks A, Jo T, Japkowicz N (2004). A multiple resampling method for learning from imbalanced data sets. Comput. Intell..

[23] Maldonado S, López J, Vairetti C (2019). An alternative SMOTE oversampling strategy for high-dimensional datasets. Appl. Soft Comput..

[24] Zhu T, Lin Y, Liu Y, Zhang W, Zhang J (2019). Minority oversampling for imbalanced ordinal regression. Knowl. Based Syst..

[25] Elkan, C. The foundations of cost-sensitive learning. In *Proceedings of International Joint Conference on Artificial Intelligence* (2001), 973–978.

[26] Iranmehr A, Masnadi-Shirazi H, Vasconcelos N (2019). Cost-sensitive support vector machines. Neurocomputing.

[27] Guo, X., Yin, Y., Dong, C., Yang, G., & Zhou, G. On the class imbalance problem. In *Fourth International Conference on Natural Computation, 2008. ICNC’08*, 2008, vol. 4, 192–201.

[28] He H, Garcia EA (2009). Learning from imbalanced data. IEEE Trans. Knowl. Data Eng..

[29] Chawla, N. V. Bowyer, K. W., Hall, L. O., Kegelmeyer, W. P. *SMOTE: Synthetic Minority Oversampling Technique*. 2011. https://arxiv.org/abs/1106.1813.

[30] Chawla NV, Lazarevic A, Hall LO, Bowyer KW, Lavrač N, Gamberger D, Todorovski L, Blockeel H (2003). SMOTEBoost: Improving prediction of the minority class in boosting. Knowledge Discovery in Databases: PKDD 2003.

[31] Blagus R, Lusa L (2013). SMOTE for high-dimensional class-imbalanced data. BMC Bioinform..

[32] Wu, D., Lawhern, V. J., & Lance, B. J. Reducing offline BCI calibration effort using weighted adaptation regularization with source domain selection. In *2015 IEEE International Conference on Systems, Man, and Cybernetics (SMC), 2015*, 3209–3216.

[33] Su, K., Hairston, W. D., Robbins, K. A. Adaptive thresholding and reweighting to improve domain transfer learning for unbalanced data with applications to EEG imbalance. In *2016 15th IEEE International Conference on Machine Learning and Applications (ICMLA)*, Anaheim, CA, 2016, 320–325.

[34] Uijlings JR, van de Sande KE, Gevers T, Smeulders AW (2013). Selective search for object recognition. IJCV.

[35] Zitnick, C. L., Dollar, P. Edge boxes: locating object proposals from edges. In *ECCV*, 2014. 2

[36] Pinheiro PO, Collobert R, Dollar P (2015). Learning to segment object candidates. NIPS.

[37] Pinheiro, P. O. Lin, T.-Y., Collobert, R., Dollar, P. Learning to refine object segments. In *ECCV*, 2016. 2

[38] Ren, S., He, K., Girshick, R., Sun, J. Faster R-CNN: towards real-time object detection with region proposal networks. In *NIPS*, 2015. 1, 2, 4, 5, 8 10.1109/TPAMI.2016.2577031 27295650

[39] Shrivastava, A., Gupta, A., Girshick, R. Training regionbased object detectors with online hard example mining. In *CVPR*, 2016. 2, 3, 6, 7

[40] Srivastava N, Hinton G, Krizhevsky A, Sutskever I, Salakhutdinov R (2014). Dropout: A simple way to prevent neural networks from overfitting. J. Mach. Learn. Res..

[41] Lin T, Goyal P, Girshick R, He K, Dollár P (2020). Focal loss for dense object detection. IEEE Trans. Pattern Anal. Mach. Intell..

[42] Szegedy, C., Vanhoucke, V., Ioffe, S., et al. Rethinking the inception architecture for computer vision. In *Proceedings of the IEEE Conference on Computer Vision and Pattern Recognition*. 2016, 2818–2826.

[43] He, K., Zhang, X., Ren, S., et al. Deep residual learning for image recognition. In *Proceedings of the IEEE Conference on Computer Vision and Pattern Recognition*. 2016, 770–778.

[44] Glorot, X., Bengio, Y. Understanding the difficulty of training deep feed forward neural networks. In *Proceedings of the Thirteenth International Conference on Artificial Intelligence and Statistics*. 2010, 249–256.

[45] Kingma, D. P., & Ba, J. *Adam: A Method for Stochastic Optimization*. https://arxiv.org/abs/1412.6980.

[46] Luciw M, Jarocka E, Edin B (2014). Multi-channel EEG recordings during 3,936 grasp and lift trials with varying weight and friction. Sci. Data.

[47] Ang KK, Chin ZY, Wang C, Guan C, Zhang H (2012). Filter bank common spatial pattern algorithm on BCI competition IV datasets 2a and 2b. Front. Behav. Neurosci..

[48] Fleiss JL, Cohen J (1973). The equivalence of weighted kappa and the intraclass correlation coefficient as measures of reliability. Educ. Psychol. Measur..

[49] Sakhavi S, Guan C, Yan S (2018). Learning temporal information for braincomputer interface using convolutional neural networks. IEEE Trans. Neural Netw. Learn. Syst..

[50] He, K., Zhang, X., Ren, S., & Sun, J. Deep residual learning for image recognition. In *Proceedings of the IEEE Conference on Computer Vision and Pattern Recognition* pp. 770–778, 2016.

[51] Huang, G., Liu, Z., Van Der Maaten, L., Weinberger, K. Q. Densely connected convolutional networks. In *Proceedings of the IEEE Conference on Computer Vision and Pattern Recognition* 4700–4708, 2017.

